# T-RexNet—A Hardware-Aware Neural Network for Real-Time Detection of Small Moving Objects

**DOI:** 10.3390/s21041252

**Published:** 2021-02-10

**Authors:** Alessio Canepa, Edoardo Ragusa, Rodolfo Zunino, Paolo Gastaldo

**Affiliations:** Department of Naval, Electric, Electronic and Telecommunications Engineering of the University of Genoa, 16145 Genova, GE, Italy; alessio.canepa@edu.unige.it (A.C.); rodolfo.zunino@unige.it (R.Z.); paolo.gastaldo@unige.it (P.G.)

**Keywords:** object detection, neural networks, surveillance, real-time

## Abstract

This paper presents the T-RexNet approach to detect small moving objects in videos by using a deep neural network. T-RexNet combines the advantages of Single-Shot-Detectors with a specific feature-extraction network, thus overcoming the known shortcomings of Single-Shot-Detectors in detecting small objects. The deep convolutional neural network includes two parallel paths: the first path processes both the original picture, in gray-scale format, and differences between consecutive frames; in the second path, differences between a set of three consecutive frames is only handled. As compared with generic object detectors, the method limits the depth of the convolutional network to make it less sensible to high-level features and easier to train on small objects. The simple, Hardware-efficient architecture attains its highest accuracy in the presence of videos with static framing. Deploying our architecture on the NVIDIA Jetson Nano edge-device shows its suitability to embedded systems. To prove the effectiveness and general applicability of the approach, real-world tests assessed the method performances in different scenarios, namely, aerial surveillance with the WPAFB 2009 dataset, civilian surveillance using the Chinese University of Hong Kong (CUHK) Square dataset, and fast tennis-ball tracking, involving a custom dataset. Experimental results prove that T-RexNet is a valid, general solution to detect small moving objects, which outperforms in this task generic existing object-detection approaches. The method also compares favourably with application-specific approaches in terms of the accuracy vs. speed trade-off.

## 1. Introduction

The recent growth of industrial applications for object detection stimulates the research community toward novel solutions. Intelligent video analysis is the core of several industry applications such as transportation [1], sentiment analysis [2], and sport [3,4].

Deep learning lies today at the core of state-of-the-art techniques for object detection, such as Faster RCNN [5], YOLO [6] and SSD [7]. Thanks to GPUs, object detection solutions based on deep learning can support real time applications; the edge-computing market now offers a variety of relatively inexpensive devices for Artificial-Intelligence (AI): microprocessors [8], hardware accelerators [9], up to complete Systems on Module (SoM), such as the Jetson series by NVIDIA [10], and machine vision cameras such as the JeVois A33 and Sipeed Maix Bit, used in [11]. These tools rely on GPUs and a collection of software optimisations to deploy computationally intensive tasks, such as AI inference, on resource-constrained hardware. Real-time object detection on embedded devices still represents a major issue, as that goal involves quite complex architectures for deep learning. In practice, one needs a trade-off between accuracy and latency to tune each method to the target scenario.

**Figure 1:**
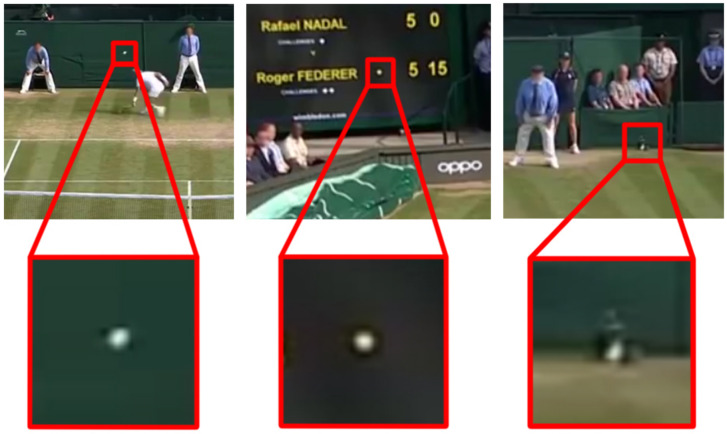
Three examples of patches which show how easily a small object might appear similar to other objects. Only the rightmost patch is a tennis ball, while the other two objects appear similar to it without actually being a tennis ball. Without a mean to discriminate the real object from potential false positives, a neural network might fail to learn how to recognise the sought object.

This paper addresses the detection of small objects, which typically take up a few tens of pixels. State-of-the-art approaches often exhibit poor performances when dealing with very small objects, due to the apparent difficulty in discriminating these features from one another and from the background [12]. Figure 1 presents an example, including three candidate sub-regions extracted from as many frames in a tennis-match video. While the rightmost frame actually includes the ball, the other patches do resemble a tennis ball but represent misclassification errors.

**Figure 2:**
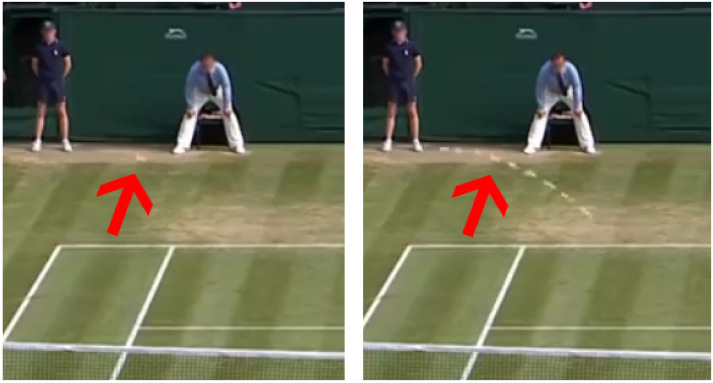
The (**left**) image shows a single frame as it is extracted from the video. The tennis ball is indicated by the red arrow and is almost undistinguishable. The (**right**) image overlays the position of the tennis ball in the previous and following frames and shows how the motion information is foundamental for its detection.

Human observers face a similar challenge when looking for tiny objects in a wide scene. The detection task, in fact, gets simpler if the target moves with respect to a still background, since the human vision system can combine motion information with the visual aspect of the object. Figure 2 clarifies this concept: the image on the left is the frame (at time $t_{n}$) drawn from the tennis video. The image on the right merges the frames from time $t_{n-5}$ up to $t_{n+3}$. In the former case, the ball is hardly distinguishable even by a human viewer, not just for its small size, but also because motion blur hinders the detection of fast-moving objects. In the rightmost image, instead, motion information makes the tennis ball clearly detectable.

The approach presented here deploys the detection of tiny moving objects in wide scenes on limited hardware resources. The method adjusts the basic building blocks of resource-constrained computer vision, and proposes a custom deep neural network for the recognition task. The T-RexNet framework improves over generic hardware-aware detectors, which only rely on visual features, and combines those features with motion information. The framework processes three consecutive frames from the video source, and prompts a set of bounding boxes around the detected objects. The overall architecture includes two stacked blocks, for feature extraction and subsequent object detection.

The dedicated pair of parallel convolutional paths in the network support that image/motion fusion process. As compared to generic object detectors, the computational overhead brought about by the two-tiered feature-extraction network is mitigated by reducing the network depth. As a matter of fact, focusing on tiny objects allows to leave out the deep layers operating at low resolution.

Single-Shot-Detector (SSD) architectures are quite popular for resource-constrained object detection. The custom feature-extraction module overcomes the well-known limitations of SSD in detecting tiny objects. The resulting feature-extraction architecture is quite shallow, and the object detection block relies on one of the least demanding available State-of-Art (SoA) solutions. In summary, the integration of these two features yields a viable solution for the real-time detection of small objects by constrained devices.

Experimental results prove that, in that context, T-RexNet improves significantly over state-of-the-art methods for generic object detection. As compared to application-specific solutions, T-RexNet exhibits a satisfactory accuracy vs/speed balance in several complex scenarios such as aerial and/or civilian surveillance and high-speed detection, tackling medium-sized to tiny objects, and varying target densities. In other words, it manages to achieve high detection rates without sacrificing accuracy too much.

The paper is organised as follows. Section 2 overviews the state-of-the-art in object detection, moving-object detection, and in the specific domains used for testing. Section 3 presents the T-RexNet approach in detail. Section 4 discusses the test scenarios considered, whereas Section 5 makes some concluding remarks. Project website with downloadable resources: http://sealab.diten.unige.it/ accessed on 8 June 2020.

## 2. Tiny Moving Object Detection: State of the Art

The identification of small moving objects is a subset of a wider research field in object detection. Existing solutions and techniques can be arranged into three main groups, namely, Single-image solutions, Background-subtraction solutions, and Spatio-temporal CNNs.

### 2.1. Single-Image General-Purpose Solutions

Typical object-detection models handle one image at a time, even when spatio-temporal information might be available. State-of-the art approaches, relying on deep learning, can be divided into region-based and single-shot detectors.

In the former models, such as R-FCN [13] and Faster R-CNN [5], a dedicated algorithm first extracts a set of Regions-of-Interest (ROIs), that is, sub-portions of the image that are likely to contain an object; then fine-detection and classification modules analyze each ROI. Single-shot detectors such as YOLOv3 [6], SSD [7] and DSSD [14], instead, avoid looping over several ROIs, and tackle the input image in a single shot.

These methods apply a library of predefined bounding boxes (anchor boxes), which have various shapes and sizes and cover the likely locations of objects in the image. The inference phase takes care of fine tuning each anchor box in terms of size and position. Region-based detectors usually prove more accurate that single-shot detectors, but are computationally demanding, as they require a loop for each single ROI [15].

In the case of small objects at low resolutions, both region-based detectors and single shot detectors tend to exhibit poor performances. Several techniques have been proposed recently to overcome that issue [16]:

- Multi-scale representation: high- and low- resolution feature maps stem from different levels of a feature-extraction network; after super-sampling low-resolution maps, features fuse together by applying either element-wise sum (Multi-scale deconvolutional single shot detector (MDSSD) [17]) or concatenation (Diverse region-based CNN (DR-CNN), [18]).
- Contextual information: the network takes into account explicitly the contextual information around a candidate object. For example, ContextNet [19] applies a custom region-proposal network specifically aimed to small objects, and for each candidate region an enlarged region is used to process contextual information.
- Super resolution: generative adversarial networks generate a higher-resolution version of the candidate object, thus improving accuracy in the detection of small objects (Perceptual generative adversarial networks (PGAN)) [20]).
- Mixed methods: features with distinct scales are extracted from different layers of a convolutional neural network; they are concatenated together, and then used to generate a series of pyramid features [21].

These methods all exhibit an increase in both computational and memory load. This brings about lower update frequency, higher latency, and ultimately might compromise implementations on resource-constrained devices for embedded applications.

### 2.2. Background Subtraction and Frame-Difference Solutions

In complex applications such as aerial surveillance, camera views can cover wide areas. Target objects (e.g., pedestrians and cars) usually span just a few tens of pixels, and the detection techniques discussed above [22] are ineffective. At the same time, in those applications the majority of input images are quasi-static and only target objects move in the scene, hence conventional background-subtraction approaches are widely adopted, even in the era of deep learning. The basic idea consists in working out the difference between a frame and the background model of the scene acquired by the same camera; the time-difference information highlights the changes caused by moving objects.

Methods differ in terms of computational cost, robustness and accuracy—Mixture of Gaussians (MOG) [23] approaches model each pixel as a random variable with a gaussian mixture model; mean-filtering [24] techniques extract the background by averaging the values of each pixel over the last N frames, whereas methods for frame-difference background subtraction [24] only consider the pixel differences between the current frame and the previous one. The latter approach is very fast but possibly less robust to noise; moreover, by disregarding any sequence of past frames, frame differences only apply when the camera is slowly moving.

Since these methods typically process gray-scale (or even B/W after threshold) images that highlight changes at a given time, the actual detection of moving objects requires some post-processing. This might possibly include morphological transformations, blob detection [25], or more complex computations [26,27,28], to the detriment of detection speed.

### 2.3. Spatio-Temporal Convolutional Neural Networks (CNNs)

The literature witnesses the growth of spatio-temporal CNNs, which take into account both visual and motion data. In MODNet [29], the authors proposed a two-stream neural network that processed input RGB images and optical flows, thus learning object detection and motion segmentation at the same time. The research presented in [30] adopted an end-to-end approach for video classification. A pseudo-3D neural network learned spatio-temporal information by considering multiple consecutive frames, which were processed by a series of convolutional filters in both the spatial (1× 3 × 3) and the temporal (3 × 1 × 1) domains. The 3D neural networks virtually replaced explicit image pre-processing steps such as background subtraction or optical-flow computation.

A spatio-temporal CNN supported the detection of vehicles in Wide Area Motion Imagery (WAMI) [31]. In the 2-stage approach, a CNN first handled 5 consecutive images (taken by an aerial surveillance system) and highlighted promising regions. The second stage completed fine detection within each region. The TrackNet approach [3] applied spatio-temporal CNNs to track small fast-moving objects in sport applications; a fully convolutional neural network could accurately track a tennis ball by processing 3 consecutive video frames (taken by a steady camera). The CNN prompted a heatmap of the possible positions of the ball, subsequent blob detection eventually yielded the predicted location.

Spatio-temporal CNNs for object detection can prove effective, but also exhibit some drawbacks: they are often computationally heavy; the various approaches are normally tailored to specific applications, and application-independent detection of small objects has not been proved yet.

### 2.4. Summary of Contribution

The methods discussed above all exhibit some features that make them unsuitable to support the Real-Time detection of small moving objects on resource-constrained devices; specific shortcomings possibly include the inability to recognize tiny objects, impractical computational loads, or lack of general applicability. The approach described in this paper can perform detection of small moving objects by maintaining some crucial features—it is lightweight and suitable for embedded devices, accuracy keeps comparable to SoA approaches and improves over them in particularly challenging conditions, the system is end-to-end trainable, and finally the method is application independent, as it performs satisfactorily in different scenarios.

## 3. Methodology

T-RexNet combines several of the techniques mentioned above to detect small moving objects in a fast, lightweight manner. The system benefits from the versatility of an end-to-end fully convolutional neural network, it processes differences between frames to involve motion information, and relies on the efficiency of MobileNet-based convolutions to integrate visual and motion data. Single-shot detectors attain real-time performances. Thus T-RexNet can be regarded as a spatio-temporal, single-shot, fully convolutional deep neural network, as per Section 2. With only 2.38 M parameters, T-RexNet turns out to be one of the most lightweight networks in the object detection field.

**Figure 3:**
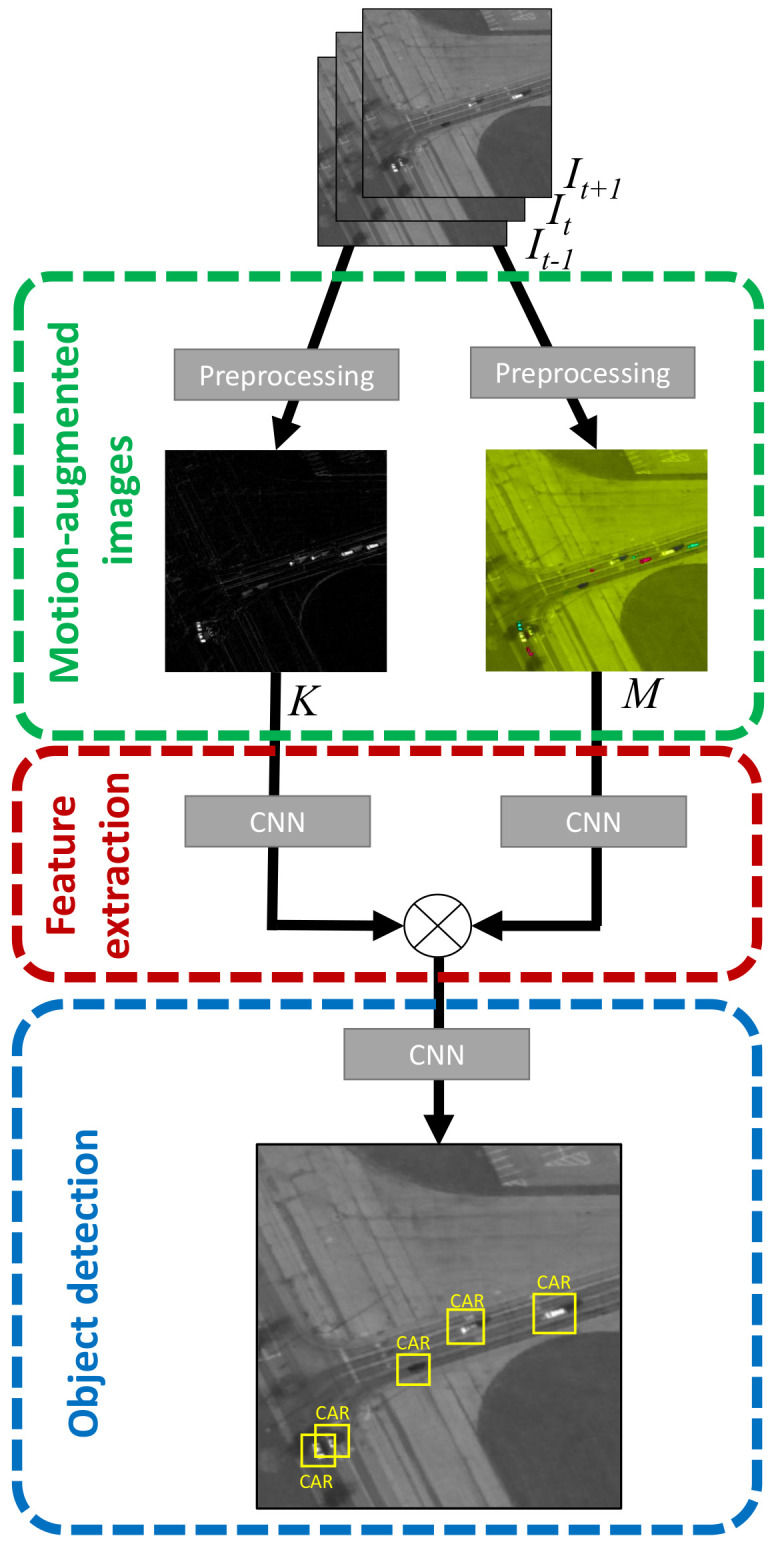
T-RexNet macro architecture, showing the two parallel “Motion-only” and “Mixed Visual-Motion” MobileNetv2-Based feature extractors. Their output is concatenated and then processed by an SSD network.

Figure 3 outlines the three-step structure of T-RexNet. Three time-consecutive gray-scale images $I_{t-1}$, $I_{t}$, $I_{t+1}$ make up the system input, where $I_{\left\{\cdot \right\}}$ denotes the 2D matrixes of pixel intensities at different time steps. The algorithm first works out a pair of motion-augmented pictures, *M* and *K*, which undergo a feature-extraction process based on two separate parallel convolutional paths. The actual object-detection results stem from the third SSD-based step.

### 3.1. Step 1: Extracting Motion-Augmented Images

This module received in input three gray-scale input frames, $I_{t-1}$, $I_{t}$, $I_{t+1}$. Since gray-scale images are represented as matrices of size [height × width × 1], stacking three of them we obtain a [height × width × 3] matrix, which is equivalent to the size of a single colored image. In other words, compared to traditional object detection methods, we substituted color with temporal data. The input of the network is processed in order to generate the pair {*M*, *K*} of motion-augmented images, as explained in the following.

**Figure 4:**
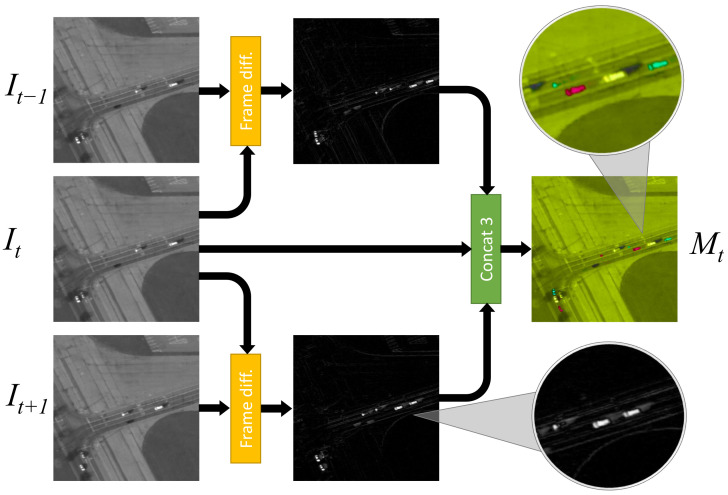
Computation of motion-augmented image *M*. For visualization purposes, after the concatenation, each of the three channels is displayed as a single color channel like in RGB images. Here, with respect to RGB, for visualization purposes the hue of the whole image has been modified. In the zoomed area of the final *M* image we can see that a moving car appears as 3 cars, corresponding to time instants t−1, *t* and t+1.

The image *M* includes three channels that are worked out as:

$$M_{t}^{1}=|I_{t+1}-I_{t}|,\;M_{t}^{2}=I_{t},\;M_{t}^{3}=\left|I_{t}-I_{t-1}\right|,$$

where the superscripts (1,2,3) refer to the channel number and the |·| is the absolute-value operator. Figure 4 illustrates the overall process in a graphic form. Channel $M^{2}$ preserves visual features, while channels $M^{1}$ and $M^{3}$ bring in motion information via frame differencing, which proves much faster than conventional background-subtraction techniques. It must be noted that preserving single-frame visual features in one of the three channels of the image makes the network able to detect, in principle, also non-moving object.

The image *K* is the concatenation of the first and the last channels of *M*, hence it only holds motion data without any visual feature.

### 3.2. Step 2: Feature Extraction

Feature-extraction networks typically include stacks of convolutional layers and pooling layers, in which lower layers involve the details of the image, whereas the topmost layers extract object-related information [32]. From a spatial point of view, the deeper is the feature map in the network, the larger is the receptive field of each of its “pixels”.

T-RexNet aims to detect small objects, hence high level information can be disregarded, and the number of stacked layers in the feature extraction network reduces accordingly. This feature also entails a beneficial effect on latency. In principle, high-level features might provide context information and therefore help localise small objects; at the same time, reducing contextual information makes the feature extractor more independent of any specific scenario and therefore maximally flexible. Feature extraction in T-RexNet involves two convolutional paths that process visual-motion mixed data (image *M*), and only motion-related data (image *K*), respectively.

**Figure 5:**
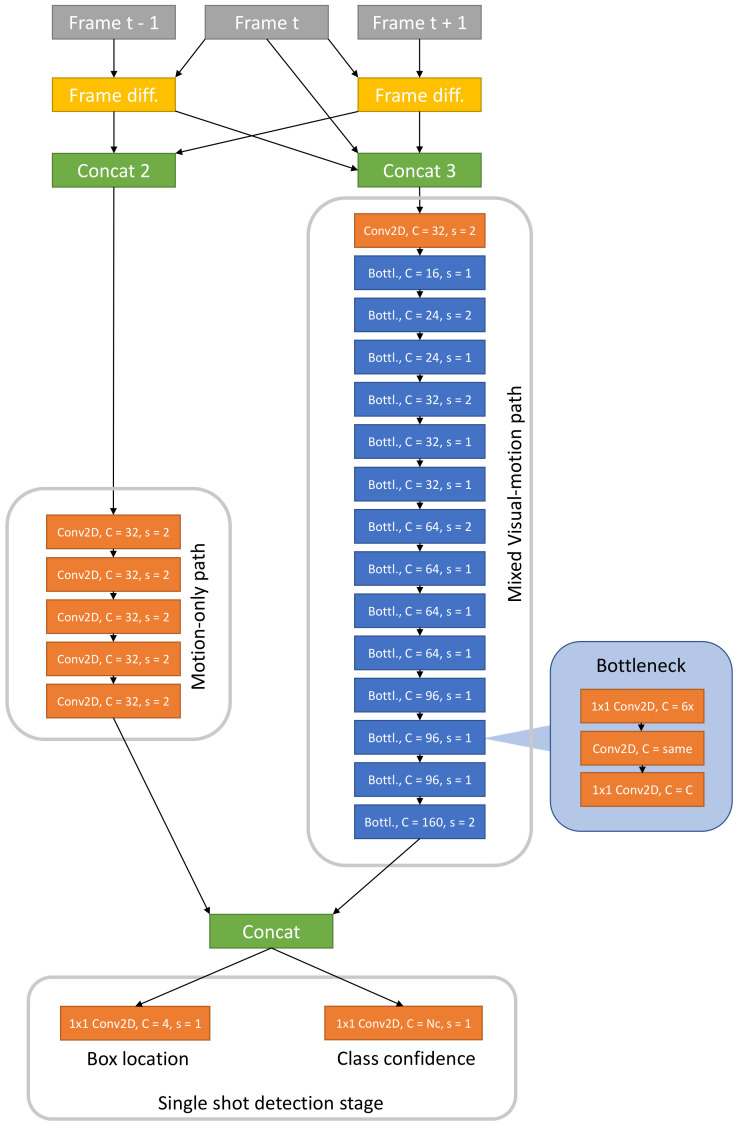
T-RexNet full architecture and image processing high level view. All the Conv2D blocks in the motion-only path use a 3 × 3 kernel. *C* is the number of output channels, *s* is the stride. Box locations are encoded with 4 numbers according to [7]. Bottleneck block [7] is highlighted: *C = 6x* means that the first block of the bottleneck is an expansion block which increments by a factor of 6 the number of channels; *C = same* means that the number of output channels is equal to the input ones; *Nc* is the number of classes.

The rightmost path in Figure 3 processes image *M* and relies on a custom network drawn from the MobileNet [33] model. This is a family of Neural-Networks (NN) architectures specifically designed for low-latency execution on mobile devices, and yields a promising balance between computational cost and accuracy. T-RexNet inherits from MobileNet the use of bottleneck residual block as a main building block, as shown in Figure 5, to limit the sensitivity to high-level, context-dependent information.

The leftmost path in Figure 3 takes into account the motion-related data held in image *K*. The architecture features a stack of several 2D convolutions, as per Figure 5. The stride is set to 2, hence the input image is downsampled to match the output resolution of the parallel convolutional path.

Finally, the outputs of the two paths are concatenated channel-wise.

### 3.3. Step 3: Object Detection

The object detection block relies on SSD [7], which mitigates computational costs as compared with region-based approaches and better fits real-time applications. Since, in the inference phase, the method prompts predictions for the whole list of predefined anchors, execution time turns out to be image independent.

Detection in the basic SSD involves several feature maps that are extracted at different levels of the feature-extraction network (the *base network* in [7]). This technique improves the robustness to different object scales. Since T-RexNet is targeted at detecting small objects, the output of the first stage just involves one feature map to contain computational costs.

T-RexNet associates each element of the feature map (i.e., each position in the map grid) with the dimension/position information and the classification (car/pedestrian/background etc.) of the corresponding anchors. The anchor size is set to 0.2·size(I), where *I* is a squared input image and size(·) is a function which returns the height and width of the image. The anchor’s aspect ratios depend on the shapes of the target objects. The standard values are {0.5, 1, 2}, which correspond, respectively, to horizontal shape, squared shape and vertical shape.

## 4. Experimental Setup

### 4.1. Scenarios

Three heterogeneous scenarios formed the test-bench for assessing the performances of T-RexNet, namely, aerial surveillance, civilian surveillance, and fast object tracking. Table 1 summarises the characteristics of the three scenarios and gives four quantities: the number of objects to be detected, the target object size, the speed of objects, and the overall image size in pixels.

To ensure fair tests, comparisons included methods with the following features:

(1) the research community proved the comparison’s effectiveness in object detection and its implementation on embedded devices; the experiments focused on each method’s ability to detect small moving objects;
(2) the various methods had been targeted to their specific test scenario, hence comparisons with T-RexNet could highlight the latter’s balance between accuracy and speed.

The Appendix A gives details about the training procedures adopted for T-RexNet, whereas the actual experimental outcomes are discussed in Section 5.

**Table 1:** Overview of the test scenarios considered in this work. Object size and speed are relative to the image frame. Image size is in pixels and measures the side of a squared image.

Scenario	# of Obj.	Obj. Size	Obj. Speed	Im. Size
Aerial surv.	High	Small	Mid	2000
Civilian surv.	Medium	Med. & small	Low	512
Fast obj. track.	Single	Small	High	300

#### 4.1.1. Aerial Surveillance

Aerial-surveillance tests addressed the Wright-Patterson Air Force Base (WPAFB) 2009 dataset, which is a well-established benchmark in Wide-Area-Motion-Imagery (WAMI). Surveillance relies on powerful camera set-ups (and software) to detect and track hundreds of targets, usually people and vehicles, possibly over areas of several squared kilometres. This typically calls for airborne systems. Targets can be so small that motion information is required to distinguish them from the background or noise. Background-subtraction techniques are therefore popular for object detection in this field [34].

The WPAFB dataset holds images taken by an airborne system and focuses on moving vehicles. Each frame roughly includes 315 million pixels and merges the shots by six, partially overlapping, gray-scale camera sensors [31]. The total area covered by each frame is around 19 squared km and the frame rate is about 1.25 Hz. On average, each target vehicle covers a region of about 100 pixels. Due to the considerable size of each raw image, state-of-the-art methods address a set of Areas of Interest (AOI); this allows fair comparisons between the various approaches [34].

The tests presented in this paper involved AOI 1, 2 and 3, as they covered a variety of layouts with different intensities of traffic. The size of each AOI was 2000 × 2000 pixels. The WPAFB dataset gave the position of a vehicle within an AOI by means of the target coordinates, (x,y); the position was mapped into a squared bounding box of 31 × 31 pixels. Stationary vehicles were not taken into account to focus on moving targets; thus cars whose positions changed less than 15 pixels between two consequent frames were removed.

T-RexNet was trained on AOIs 1 and 3, which covered high-intensity and low-intensity traffic situations, respectively. The images were taken from a moving camera, and the test phase involved the remaining set, AOI 2. To ensure fair comparisons with other approaches, time-consecutive images were recorded to support frame differencing. Due to the excessive size of input images (for T-RexNet as well as other object detectors), in compliance with the approach [35] each 2000 × 2000 image was split into a set of 16 partially overlapping pictures, each holding 512 × 512 pixels. To be consistent with the literature [34], true positives were only considered when the center positions of the detected boxes lied within a 20-pixel distance from the ground-truth location.

#### 4.1.2. Civilian Surveillance

The CUHK Square dataset [36] addressed people detection, and included videos recorded (@30 Frames-Per-Second, FPS) by a surveillance camera monitoring a square and a road crossing. Spatial resolution was 720 × 576 pixels. Since the original dataset featured some misdetections [1], the proper labels were manually added. The overall set of videos included 2105 detections for training and 593 detections for testing.

The quasi-horizontal inclination of the camera affected the depth of the scene and the perspective; as a consequence, people close to the camera appeared much bigger than people on the background. Thus CUHK also allowed to test the effectiveness of T-RexNet in detecting medium-sized objects.

At 30 FPS, the slow advance of walking people resulted in minimal changes between time-consecutive frames, hence the input videos were downsampled to 1 Frame-Per-Second (FPS), and the spatial resolution was resized to 512 × 512 pixels. That downsampling factor set the trade-off between the amount of motion data captured and the update frequency of the detections.

The experiments only considered valid detections when an IoU exceeded the 50% threshold with respect to the ground truth. Whenever a bounding box was associated with multiple ground truth points, the tests only considered one candidate, on a minimum-distance basis.

#### 4.1.3. Tennis Ball Tracking

This setup only included one target object per frame. The example presented in Figure 2 points out the difficulty of tennis ball tracking in real-time detection: the small size of the ball and the motion blur caused by its fast movement made it almost undetectable even by human observers without the aid by motion data.

**Figure 6:**
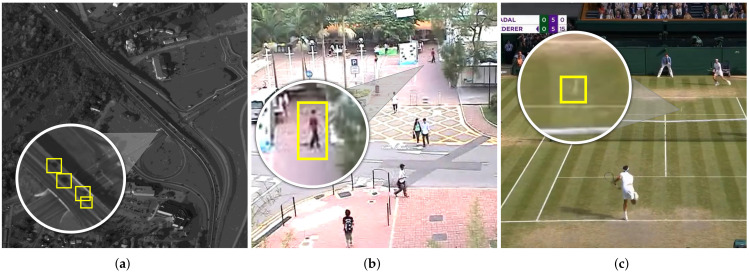
The three test scenarios considered in this work: (**a**) aerial surveillance, WPAFB 2009 dataset; (**b**) civilian surveillance, CUHK dataset; (**c**) Tennis ball tracking, custom dataset. In each image a sub-area is zoomed to highlight the small size of the target objects.

Tennis ball tracking lacks publicly available benchmarks, hence the training set collected 18,220 labeled frames extracted from videos of various matches and recorded at 30 FPS. To avoid overfitting, the test set included 5160 frames taken from three videos taken in different courts and with the camera placed at different heights, as per Figure 6c. In the following, we will refer to these videos as Court A, Court B, and Court C. In both the training and test set, the ground-truth labels were generated by using TrackNet [3], whose precision, according to the authors and to our observations, exceeded 95%. The result of such an automatic labelling method was anyway checked manually for correctness.

This scenario aimed to assess the suitability of T-RexNet for fast, real-time detections; in both the training and the test set, the frames were downsampled to 300 × 300 pixels. Only the detected boxes whose center was closer than 16 pixels to the ground truth location were considered as true positives.

### 4.2. Deployment

To allow a fair comparison with other methods in the literature and make the repeatibility of our experiments easier, we first performed our tests on a Desktop PC provided with an NVIDIA GTX 1080 Ti graphic card.

Then, to prove the suitability of our method to embedded edge-AI devices, we deployed it on an NVIDIA Jetson Nano [37], using its development board. This is the more resource constrained device of the NVIDIA Jetson series, a suite of hardware platforms specifically designed for bringing Artificial Intelligence to the edge. It is a System-On-Module (SoM) which features HW acceleration for deep learning and runs a proprietary modified version of Ubuntu 18.04. Basic characteristics of the SoM (not including development board) are reported in Table 2.

Users can set hardware utilization using a software interface. Two optimized configurations called 5 W and Max-N are available. The first one limits power consumption setting a clock frequency of CPU and GPU to 0.90 and 0.64 GHz, respectively. In addition, two cores of the CPU are turned off. In Max-N configuration all the hardware resources are set to maximize performance, at the expense of power consumption.

NVIDIA provides a toolchain based on TensorTRT. This tool provides an optimized implementation of common deep learning layers for Jetson devices. For the case of TF models, the output of TensorTRT is again a TF frozen graph where the computed layers are replaced with optimized versions. TensorTRT can adopt different data sizes when deploying a network: standard floating-point representation (FP32), half-precision floating point (FP16) and 8-bit integer representation (INT8). The experiments were conducted with the FP16 format since this provides a good trade-off between accuracy and power consumption [38]. In addition, the results proved that FP16 is indeed sufficient to reach good frame rates using Jetson Nano.

The code was developed in Python using the CV2 module and TensorFlow. The computed latency considered only network processing. Each frame was elaborated in real-time when acquired without the use of any batching strategy. The measures involved 100 images. The tests involved two versions of T-RexNet, one with input size 300 × 300 and another with input size 512 × 512. The networks were optimized using tensorRT with FP16 representation. The results measured the average inference time for optimized and non-optimized models, using the Jetson Nano with different power settings and different input sizes.

## 5. Results

This section illustrates the results of the experiments performed in the three test scenarios. According to our previous findings [38] we observed that moving the same architecture from the Desktop to the embedded platform has negligible impact on the detection accuracy, while it mostly affects speed and memory footprint. Therefore, in Section 5.1, Section 5.2 and Section 5.3 we first illustrate, for each scenario, our achievements using the Desktop platform and, then, in Section 5.4, we analyze the impact of deploying T-RexNet on the Jetson Nano.

Table 3, Table 4 and Table 5 give an overview of the comparisons, in terms of F1 scores, with existing methods in the literature.

### 5.1. Aerial Surveillance

**Figure 7:**
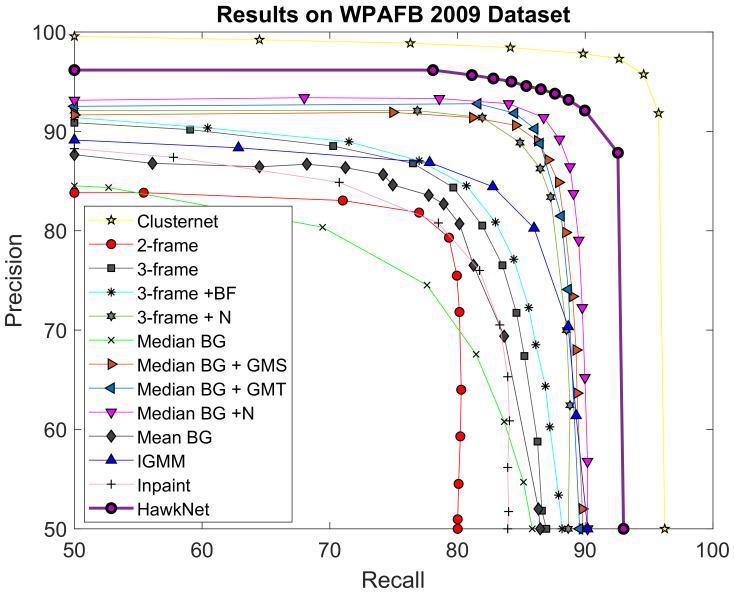
Comparison between the results achieved by our T-RexNet and other State-of-Art (SoA) approaches in the aerial surveillance (WAMI) scenario. We took the comparison in [31] and added the results of using T-RexNet. Despite Clusternet has better performance, our T-RexNet is ∼10 times faster. For information about the other methods we compare with refer to [31].

Figure 7 shows the ROC curves achieved by T-RexNet and other state-of-the-art algorithms on the AOI 2 test set. The ROC curve for T-RexNet was added to the original plot reported in [31]. To assess the balance between recall and precision the experiments applied various threshold values on the detection confidence. We remind the reader that Recall = TP/(TP+FN); Precision = TP/(TP+FP); where TP, FP, FN are True/False Positives/Negatives.

T-RexNet outperformed the other comparisons in terms of accuracy, with the exception of ClusterNet [31], which scored near-optimal performances. As reported in [31], however, ClusterNet required 2–3 s per image on a Titan X GPU board, depending on the number of selected regions; a time span of 3 s covered the inference phase to inspect the whole image for fine detection. By contrast, the inference time for T-RexNet was 310 ms per image on our Desktop platform featuring an NVIDIA GTX 1080 Ti board, which is similar to a Titan X in terms of hardware resources and computational performances.

### 5.2. Civilian Surveillance

**Figure 8:**
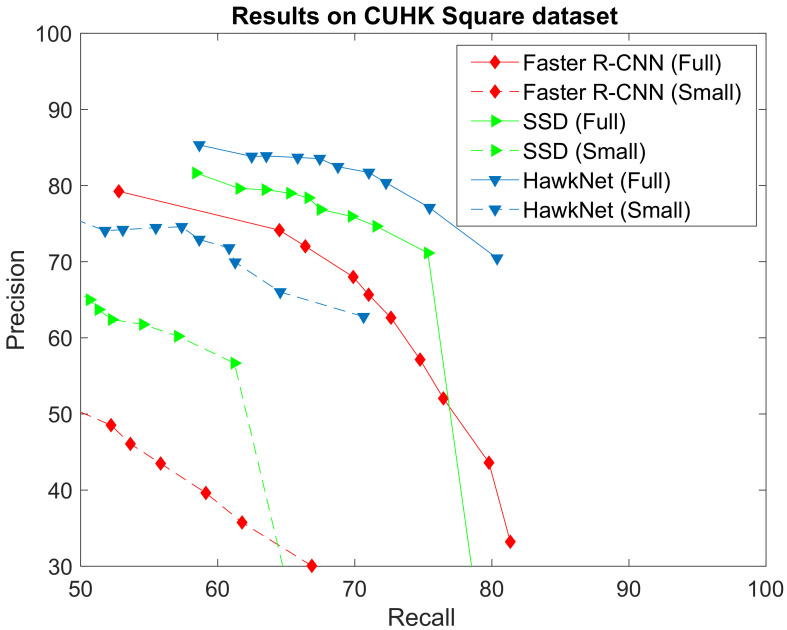
Comparison between the results achieved by our T-RexNet, SSD and Faster-RCNN in the civilian surveillance test case using the CUHK square dataset. Full and Small indicate whether the test has been conducted over the whole image or the upper half only, where perspective makes people much smaller and the gap between our approach and others is even more pronounced.

Figure 8 gives the ROC curve scored by T-RexNet in object detection within one image. The obtained results are compared with the corresponding curves attained by SSD [7] (with MobileNetv2 [33] as backbone network) and Faster R-CNN [5] (with ResNet50 [39] as backbone network). The Figure gives two curves for each comparison: the Full mark refers to experiments on whole images, whereas Small curves refer to tests only performed on the upper halves of images, where perspective made people appear smaller.

The ROC curves in Figure 8 witness that motion information greatly helped T-RexNet achieve the best performance. More, T-RexNet was the only architecture that attained satisfactory results when focusing on tiny objects.

### 5.3. Tennis Ball Tracking

**Figure 9:**
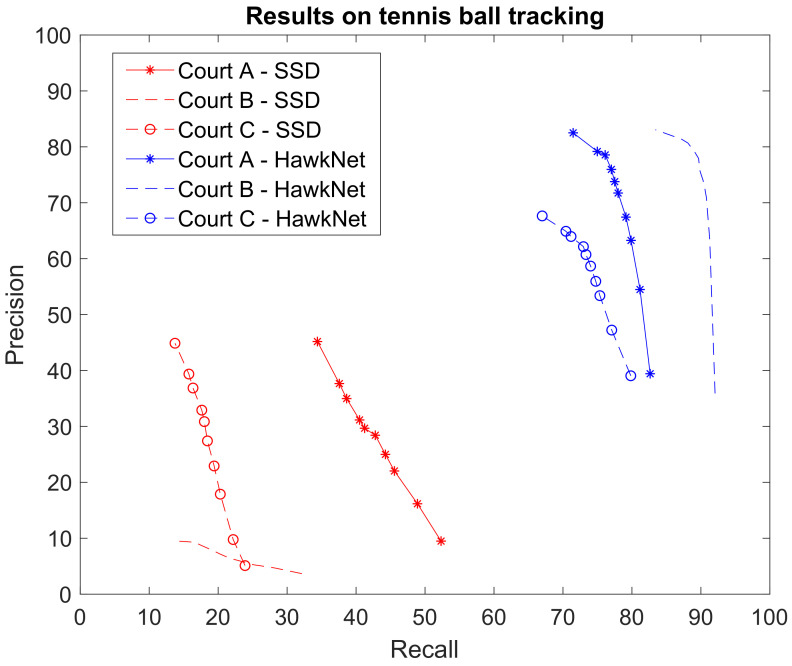
Comparison between the results achieved by our T-RexNet and SSD in the tennis ball tracking test case. Due to the motion blur and the small scale of the ball, it becomes almost undetectable by SSD, since it does not exploit motion data.

Figure 9 shows the ROC curves measured by applying T-RexNet on the test sets Court A, Court B, and Court C. The graph also give the associate ROC curves obtained by MobileNetv2-SSD, which represented the single-image architecture from which T-RexNet evolved. The comparison pointed out the significant impact of involving motion data in the detection of the target object.

Experimental outcomes prove that T-RexNet featured a remarkable improvement over State-of-the-Art, application-independent approaches. When considering application-specific solutions, TrackNet [3] had generated our ground-truth labels and proved more accurate than T-RexNet in tennis-ball tracking. As reported in the original paper, TrackNet attained on average higher F1 scores than 0.84, which was consistent with the test performed in this research. At the same time, TrackNet proved significantly heavier than T-RexNet: Python implementations of both, running on the Desktop platform, resulted in 2. 2 FPS for TrackNet and 47 fps for T-RexNet, that is ∼21 times faster. The limited resolution of input images allowed to increase the batch size in the inference phase up to 10 consecutive frames, while still fitting the memory of the test GPU. This batch approach allowed T-RexNet to run at 96 fps, at the price of an increased latency from 21 ms to 104 ms.

### 5.4. Deployment of T-RexNet on the Jetson Nano

This section presents the results of the deployment on Jetson Nano. Table 6 shows on the rows the power setting of the board. Columns are divided into couples. The first pair reports the result for input size 512 × 512, the second refers to 300 × 300. The first column of each pair refers to an optimized model with FP16 representation. The second column indicates the original TF model.

The results reveal that T-RexNet can be deployed in embedded systems with real-time performances. In Max-N configuration, the network can process a frame in 70.28 ms. In other words, the device could elaborate 13 FPS, which is acceptable for many applications. The comparison with native TensorFlow solutions highlights the importance of optimization combined with FP16. A similar observation holds for 5W power mode.

Memory requirements for this network are quite limited. The *pb* file, that is the TensorFlow’s ProtoBuf file containing the description of the network, measures around 3.0 MB. The memory strategy implemented on Jetson Nano allocates a large amount of memory that is not directly dependent on the model size. Accordingly, a direct measure would yield biased results. Indeed, literature proves that similar models can be deployed in devices using a smaller memory footprint [38].

## 6. Conclusions

The T-RexNet approach involves a deep neural network for the detection of small moving objects. It uses motion data as a discriminant contribution whenever visual-only information is limited due to the small target sizes. The T-RexNet architecture includes a two-path network, while keeping computational costs low. The method’s relevant features consist in limiting computational and memory costs, allowing real-time execution, ensuring reuse in several applications with an end-to-end approach, and yielding remarkable accuracy performances that favourably compare with SoA approaches. T-RexNet was tested in three real-world scenarios covering a wide range of applications. Accuracy results confirmed that the proposed method outperformed most of SoA approaches; conversely, when considering execution speed, T-RexNet improved over the most accurate methods. Tests performed on an NVIDIA Jetson Nano proved that our solution is suitable for deployment on embedded edge devices. In conclusion, we believe T-RexNet can be regarded as an easy-to-use alternative, suitable for embedded to high-end devices, to deal with tiny moving targets. 

## Figures and Tables

**Table 2:** Characteristics of the NVIDIA Jetson Nano System-on-Module.

Parameter	Value
AI Performance	472 GFLOPs
GPU	128-core NVIDIA Maxwell GPU
CPU	Quad-Core ARM Cortex-A57 MPCore
Memory	4 GB 64-bit LPDDR4 25.6 GB/s
Storage	16 GB eMMC 5.1
Power	5 W/10 W
Mechanical	69.6 mm × 45 mm 260-pin SO-DIMM connector

**Table 3:** Comparison of F1 scores achieved in the Aerial surveillance scenario. Numbers in brackets represent the measured speed, in frames per second, with the Desktop platform. The asterisk indicates that the number is retrieved from the original paper.

	Aerial Surveillance
**T-RexNet **	0.91 (3)
**ClusterNet**	0.95 * (0.3 *)
**Median BG+ N**	0.89 *

**Table 4:** Comparison of F1 scores achieved in the Civilian surveillance scenario. Numbers in brackets represent the measured speed, in frames per second, with the Desktop platform. This scenario is splitted into the sub-cases of normal and small object size to highlight the results of our method when objects are particularly small.

	Civilian Surveillance	
	**Normal**	**Small**
**T-RexNet**	0.77 (44)	0.79 (44)
**Faster R-CNN**	0.69 (23)	0.5 (23)
**SSD512**	0.73 (41)	0.59 (41)

**Table 5:** Comparison of F1 scores achieved in the Tennis ball tracking scenario. Numbers in brackets represent the measured speed, in frames per second, with the Desktop platform. This scenario is splitted into the three videos we considered, with different camera view, court and environment. The asterisk indicates that the number is retrieved from the original paper.

	Tennis Ball Tracking		
	**A**	**B**	**C**
**T-RexNet**	0.78 (47)	0.84 (47)	0.67 (47)
**SSD300**	0.34 (43)	<0.2 (43)	0.23 (43)
**TrackNet**	>0.84 * (2.2)	>0.84 * (2.2)	>0.84 * (2.2)

**Table 6:** Inference time measured on the NVIDIA Jetson Nano device for every combination of image size, power mode and optimization level.

Power Mode	512 × 512		300 × 300	
	TRT (ms)	TF (ms)	TRT (ms)	TF (ms)
Max-N	70.28	437.15	65.45	431.28
5W	108.77	616.74	98.28	661.14

## Data Availability

Project website with downloadable resources: http://sealab.diten.unige.it/ accessed on 8 June 2020.

## Appendix A. Hyperparameters and Training Details

In this section we use S1, S2, S3 to denote our three scenarios: aerial surveillance, civilian surveillance, tennis ball tracking.

T-RexNet has been implemented in Python, version 3.6.9, using the TensorFlow library, version 1.14. All the models have been trained from scratch using an NVIDIA GTX 1080 Ti graphic card. The batch size has been set to 32, and the number of training steps to 20.000 in S1 and S2, and 60.000 in S3. RMSprop optimizer has been used, with a learning rate of 0.004, momentum and decay equal to 0.9. Hard negative mining was used to balance positive vs. negative (background) classes, with a maximum of 3 negative per positive examples. All the convolutions use L2 regularization with a weight of 0.00004. No dropout has been implemented. The default size of the anchor boxes has been set to 0.2 times the size of the image, while the aspect ratio has been choosen on a scenario basis: 1 in S1 and S3, 2 in S2. Training time lasted approximately 10 h per scenario.
